# Prevalence and capsular type distribution of *Streptococcus agalactiae* isolated from pregnant women in Namibia and South Africa

**DOI:** 10.1186/s12879-019-3809-6

**Published:** 2019-02-20

**Authors:** Munyaradzi Mukesi, Benson C. Iweriebor, Larry C. Obi, Uchechukwu U. Nwodo, Sylvester R. Moyo, Anthony I. Okoh

**Affiliations:** 1 SAMRC Microbial Water Quality Monitoring Centre, University of Fort Hare, Private Bag X1314, Alice, Eastern Cape Province 5700 South Africa; 2 Applied and Environmental Microbiology Research Group (AEMREG), Department of Biochemistry and Microbiology, University of Fort Hare, Private Bag X1314, Alice, Eastern Cape Province 5700 South Africa; 3 Department of Health Sciences, Faculty of Health and Applied Sciences, Namibia University of Science and Technology, Private Bag, Windhoek, 13388 Namibia; 4 Academic and Research Division, University of Fort Hare, Private Bag X1314, Alice, Eastern Cape Province 5700 South Africa

**Keywords:** Prevalence, Capsular type, *Streptococcus agalactiae*, Pregnant, Women

## Abstract

**Background:**

*Streptococcus agalactiae* or Group B *Streptococcus* (GBS) is the leading cause of neonatal morbidity and mortality resulting in septicaemia, bacteraemia and meningitis. Long term problems in children range from loss of hearing to mental retardation. While Intrapartum Antibiotic Prophylaxis (IAP) has reduced the incidence of *S. agalactiae* infection, it still remains the leading cause of disease in neonates. GBS has ten capsular types whose distribution varies across the world. Therefore, this study sought to determine the prevalence of GBS in Namibia and South Africa amongst pregnant women between 35 and 37 weeks gestation and elucidate the capsular types.

**Methods:**

Lower vaginal and rectal swabs were collected from pregnant women between 35 and 37 weeks gestation. Five hundred and thirty pregnant women were recruited into the study in Windhoek, Namibia while one hundred pregnant women were recruited in the Eastern Cape, South Africa. The swabs were cultured on 5% sheep blood agar (Biomerieux, New Jersey, USA) for isolation of GBS. Presumptive isolates were confirmed using both the Vitek (2) and molecular techniques targeting the *scp*B gene. Capsular typing was performed in a multiplex PCR with capsular specific primer pairs.

**Results:**

The prevalence of GBS in Namibia was 13.6 and 37% in South Africa respectively. In both countries most women were dually colonised with GBS. Capsular types II, III and V were the most prevalent.

**Conclusions:**

The prevalence of GBS in Namibia was lower than in South Africa in this study. The prevalence in both countries was not different from those reported in other African countries and around the world. The predominant capsular types in this study are the ones commonly associated with adverse maternal outcomes.

## Background

*Streptococcus agalactiae* also known as Group B *Streptococcus* (GBS) is a gram positive bacteria which is the sole organism in Lancefield group B. The organism was first described in 1887 as a cause of bovine mastitis but was only noted as a human pathogen in 1935 [1] and it has been isolated from many other animals including fish, dogs, horses and guinea pigs [2].

GBS primarily colonizes both the gastrointestinal and genital tracts but can also be found in the oropharynx. In the 1970s, GBS emerged as the leading cause of neonatal sepsis and meningitis with case fatality rates of up to 50% and since then, it has remained the leading cause of neonatal sepsis and meningitis in the United States [3]. The pathogenesis of these infections is based on GBS colonization of the mother vaginally or rectally and on transmission of the organism during labour or delivery [4]. Colonization rates vary between countries but as many as 20–40% of healthy women are asymptomatically colonized and are at risk of transmitting it to their new-born babies [5]. It can cause serious disease in neonates, pregnant women and immune-compromised patients. In pregnant women the disease presents as infection of the genital tract, placenta, amniotic sac or simply bacteraemia. While infection does not cause death in pregnant women, in 10–60% of the cases it results in either miscarriage or stillbirth in developing countries when compared to with 7–11% in developed countries [6, 7].

Vertical transmission of GBS from colonized mothers to their new-borns can result in early onset GBS infection which occurs in the first 7 days of life and is a leading cause of invasive bacterial infection in neonates. Mortality in early onset disease is estimated at 5% and is characterized by bacteraemia, pneumonia and meningitis [8, 9]. Mortality is higher in preterm infants with case fatality rates of up to 20% and as much as 30% in those born before 33 weeks of gestation [5]. Late onset disease refers to infections from one week to 90 days of age and this is attributed primarily to transmission after birth from the mother or other external sources to the neonate. Late onset disease presents primarily with meningitis and associated sequelae such as deafness and developmental disabilities [10].

Surface associated polysaccharides (capsules) are common in both gram positive and gram negative bacteria. Microorganisms develop capsules for protection against environmental factors and survival against the host’s defence mechanisms. In human beings, it allows the microorganisms to escape phagocytosis, complement mediated killing and acquired immune responses by masking bacterial antigenic determinants, mimicking host antigens and interfering with complement mediated killing. However, the capsule can also act as a target for specific antibodies, conferring the host with immunity to GBS infection [1].

Capsular serotyping has been one of the mainstays in the descriptive epidemiology of GBS as currently ten capsular serotypes (1a, 1b, 11-IX) have been described based on the antigenicity of their capsular polysaccharides. These capsules represent one of the major virulence factors of GBS and of these, serotype III has been noted to be responsible for the majority of GBS infections including meningitis in neonates [11]. Recent epidemiological studies have pointed out to greater involvement of serotype V, now accounting for GBS disease in approximately 30% of non-pregnant adults and 14 to 23% of pregnant women and neonates [12]. Other studies have reported GBS serotypes Ia, Ib, II, III and V as being responsible for most early onset disease [13, 14]. Association of serotypes with disease might have to do with sialic acid residues incorporated in the capsular polysaccharide primary structure.

Capsular polysaccharides are composed of repeating units of four to seven monosaccharides with a backbone and side chains [15]. Of the ten serotypes, eight are closely related genetically and structurally with serotype VIII being distantly related. This suggests that despite the evolutionary pressures toward antigenic variation exerted by hosts’ acquired immune responses, GBS capsular polysaccharides still remain highly conserved.

Capsular polysaccharide distribution varies even within geographical areas. In South Africa in 2011, serotype III was found to be common among mothers (37.3%) and new-borns (36.2%). Serotypes Ia, Ib and III were more prevalent among pregnant women (74.1%) and new-borns (69.6%) with serotypes III and Ia causing 53.9% of invasive disease in infants aged 7 to 90 days as compared to Ib, II, IV and V (< 6%) [16]. A study carried out on pregnant women in Zimbabwe in 2000 reported serotypes III and V as the most common [17] and in a recent study on pregnant women in Zimbabwe, Mavenyengwa et al detected serotypes Ia, Ib, II, III and V [18].

Studies in Malawi [19], Finland [20], Sweden [21], England [22], United States [23], Zimbabwe [18], Gambia [24], Egypt [25] and in Brazil [26] have shown variations in serotype distribution around the world. This study therefore sought to determine the prevalence of GBS and capsular type distribution of isolates colonising pregnant women in Namibia and South Africa.

## Methods

### Sample collection, culture and identification of GBS

Pregnant women between 35 and 37 weeks gestation were recruited into the study on a voluntary basis. Participants targeted were those attending Windhoek Central Hospital maternity clinic, Windhoek (Namibia) (which is urban) while in the Eastern Cape, samples were collected from Mduntsane, Dimbazi and Middle Drift clinics (which is a rural setting). Pregnant women on antibiotic treatment or who had been on treatment seven days prior to recruitment in the study were excluded. In Windhoek, out 610 women approached 530 (86.9%) took part while in South Africa the recruitment rate was 100%. A low vaginal swab (LVS) and rectal swab (RS) were collected from each consecutive participant who consented to participate. Samples were placed in Amies transport medium (Rochelle chemicals, South Africa), bar-coded for identification, placed in a cooler box containing ice packs and transported to the laboratory. In Namibia, samples were transported to the Microbiology laboratory, Faculty of Health and Applied Sciences, Namibia University of Science and Technology (NUST) for culture and presumptive identification of GBS while samples collected in South Africa were processed at the Applied and Environmental Microbiology Research Group (AEMREG) laboratory, Department of Microbiology and Biochemistry, University of Fort Hare (UFH). In both South Africa and Namibia samples were transported and cultured within 2 h of collection. Molecular confirmation and characterization was done at AEMREG laboratory.

Samples were inoculated onto Columbia blood agar containing 5% sheep blood (Biomerieux, New Jersey, USA) and incubated at 37 °C for 18 h under 5% carbon dioxide atmosphere. Sterility of the media was established by incubating a representative of the media for 2–5 days at 35–37 °C and plates were checked for no evidence of bacterial growth after incubation. Quality control was performed using the ATCC reference strain BAA-2674. Presumptive isolates were identified as GBS based on: β-haemolysis on Columbia blood agar containing 5% sheep blood (Biomerieux, New Jersey, USA), gram positive cocci in chains under the microscope after Gram staining, negative catalase reaction, Lancefield grouping with type B antisera (Becton Dickinson, New jersey, USA) and using the Vitek (Biomareux) version 2. All presumptive isolates were stored in 30% glycerol stocks at -80 °C until further analyses.

### Molecular confirmation of GBS strains

Presumptive isolates in glycerol stocks were resuscitated in Todd Hewitt broth (Biomerieux, New Jersey, USA) for 24 h at 37 °C and streaked onto Columbia blood agar containing 5% sheep blood. GBS isolates were confirmed by molecular techniques using a pair of primers specific for the *scp*B gene. A single colony of GBS was picked from Columbia blood agar containing 5% sheep blood (Biomerieux, New Jersey, USA) and emulsified with 2 mL of nucleic acid free water in a 2 mL microcentrifuge tube. The tube was boiled at 100 °C for 15 min on a Heating Block and thereafter centrifuged at 10,000 rpm (rpm) for 5 min and supernatant containing GBS DNA was separated from the pellet and stored at -80 °C. Twelve microliters of One Taq^R^ Master Mix with standard buffer (New England Biolabs, United Kingdom) containing: 20 mM Tris-HCl, 1.8 mM MgCl_2_, 22 mM NH_4_Cl, 22 mM KCl, 0.2 mM dNTPs, 5 % glycerol, 0.06% IGEPAL^R^ CA-630, 0.05 % Tween^R^ 20 and 25 units/mL One Taq DNA polymerase, 6 uL of water of PCR grade, 1 uL of 10 pMol of both forward and reverse primers were mixed with 5 μL of DNA template to make up a total reaction volume of 25 uL. *Scp*B primer sequences are presented in Table 1.

**Table 1:** Oligonucleotide primers for molecular confirmation of GBS

Name	*scp*BF
Sequence	ACAACGGAAGGCGCTACTGTTC
Name	*scp*BR
Sequence	ACCTGGTGTTTGACCTGAACTA

(Adopted from Elbaradie et al., 2009) [37]

The cycling conditions were as follows: an initial denaturation of 94 °C for 4 min followed by 35 cycles of denaturation at 93 °C for 1 min, annealing at 57 °C for 1 min and extension at 72 °C for 1 min and a final elongation step of 72 °C for 7 min followed by a hold at 4 °C as described by Desjardins et al., (2004) [27]. The following ATCC reference strains (and respective capsular type) were used as positive controls during capsular typing: BAA-1138 (Ia), BAA-1174 (Ib), BAA-2675 (II), BAA-2674 (III), BAA-2673 (IV), BAA-2672 (V), BAA-2671 (VI), BAA-2670 (VII), BAA-2669 (VIII) and BAA-2668 (IX). Procedure (negative) controls were used during PCR to detect non-specific amplification.

Two microliters of amplicons were loaded on 1 % agarose gel stained with 10 μL ethidium bromide and electrophoresed for 45 min at 110 °C in a 0.5X Tris/Borate/EDTA (TBE) buffer. Amplification was verified in a gel documentation system and photographed.

### Determination of capsular types of the isolates using multiplex PCR

Table 2 presents the oligonucleotide primers used for capsular typing. The capsular types were grouped according to annealing temperature as follows:

**Table 2:** Oligonucleotide primers for capsular typing

Primer name	Sequence	Band size (bp)
Ia-F Ia-R	GGTCAGACTGGATTAATGGTATGC GTAGAAATAGCCTATATACGTTGAATGC	521
Ib-F Ib-R	TAAACGAGAATGGAATATCACAAACC GAATTAACTTCAATCCCTAAACAATATCG	770
II-F II-R	GCTTCAGTAAGTATTGTAAGACGATAG TTCTCTAGGAAATCAAATAATTCTATAGGG	397
III-F III-R	TCCGTACTACAACAGACTCATCC AGTAACCGTCCATACATTCTATAAGC	1826
IV-F IV-R	GGTGGTAATCCTAAGAGTGAACTGT CCTCCCCAATTTCGTCCATAATGGT	578
V-F V-R	GAGGCCAATCAGTTGCACGTAA AACCTTCTCCTTCACACTAATCCT	701
VI-F VI-R	GGACTTGAGATGGCAGAAGGTGAA CTGTCGGACTATCCTGATGAATCTC	487
VII-F VII-R	CCTGGAGAGAACAATGTCCAGAT GCTGGTCGTGATTTCTACACA	371
VIII-F VIII-R	AGGTCAACCACTATATAGCGA TCTTCAAATTCCGCTGACTT	282

(Adopted from Poyart et al., 2007) [38]

Types 1a, 1b, II &III at 58 °C; IV, V, VI & VII at 59 °C and VIII at 56 °C.

Twelve microliters of One Taq^R^ Master Mix with standard buffer (New England Biolabs, United Kingdom) containing: 20 mM Tris-HCl, 1.8 mM MgCl_2_, 22 mM NH_4_Cl, 22 mM KCl, 0.2 mM dNTPs, 5% glycerol, 0.06% IGEPAL^R^ CA-630, 0.05% Tween^R^ 20 and 25 units/mL One Taq DNA polymerase, was mixed with 6 μL of nuclease free water, I uL each of 10 pMol of reverse and forward primers for the respective capsular types, 5 uL of DNA template to make a final reaction volume of 25 uL.

The cycling conditions were as follows: 94 °C for 4 min as an initial denaturation followed by 35 cycles of denaturation at 93 °C for 1 min, annealing at the respective annealing temperature for 1 min and extension at 72 °C for 1 min with a final elongation step of 72 °C for 7 min followed by a hold at 4 °C. Amplification was verified in a 1% agarose gel stained with ethidium bromide and electrophoresed at 120 V for 45 min in a 0.5X TBE buffer and thereafter viewed in a transilluminator and photographed.

## Results

Out of the 530 pregnant women recruited in Namibia, 72(13.6 %) were colonized with GBS vaginally, rectally or both while in South Africa, out of 100 women screened, 37 (37%) were colonized with GBS. All the isolates were confirmed as GBS by molecular techniques. The frequency of distribution of GBS in Namibia and South Africa is shown in Tables 3 and 4 while that of the capsular type in both countries are shown in Tables 5 and 6 respectively.

**Table 3:** Frequency distribution of GBS in Namibian isolates according to colonization site

Colonization site	Frequency (%)
Vagina only	27 (37.5)
Rectum only	13 (18.1)
Dual colonization	32 (44.4)
Total	72 (100)

**Table 4:** Frequency distribution of GBS in Namibian isolates according to colonization site

Colonization site	Frequency (%)
Vagina only	5 (13.5)
Rectum only	2 (5.4)
Dual colonization	30 (81.1)
Total	37 (100)

**Table 5:** Frequency distribution of capsular types among pregnant women in Namibia

Capsular type	Frequency (%)
II	69 (60.0)
III	29 (25.2)
V	12 (10.4)
Ia	3 (2.6)
IV	2 (1.7)
1b	0 (0)
Total	115 (100)

**Table 6:** Frequency distribution of capsular types among pregnant women in South Africa

Capsular type	Frequency (%)
II	35 (52.2)
III	12 (17.9)
V	11 (16.4)
Ia	6 (9.0)
IV	1 (1.5)
1b	2 (3.0)
Total	67 (100)

## Discussion

The prevalence of GBS colonization varies around the world according to different geographical locations as shown in studies on the prevalence of GBS conducted on both non-pregnant and pregnant women [28, 29]. The prevalence of GBS among pregnant women between 35 and 37 weeks gestation in Windhoek was 13.6% which is low compared to findings of other Southern African countries like South Africa (30.9% & 23%) in 2015 and 2016 and Zimbabwe (21–47%) in 2010 [18, 30, 31]. This study was based on an urban population and GBS colonization has been shown to be dependent on factors like sanitation and educational levels of pregnant women.

In South Africa, this current study showed a GBS prevalence of 37% in the Eastern Cape which is much higher than the prevalence in Windhoek (Namibia) and other South African studies done by Chukwu et al., (2015) and Cools et al., (2016) [30, 31]. Sampling of different populations could contribute to differences in prevalence rates of GBS colonization as a similar study in Zimbabwe in 2006, reported a prevalence of 60% in a rural population and 46% in an urban population [32]. Factors which affect GBS colonization include ethnicity, maternal age, parity, marital status and educational level and these factors vary between rural and urban populations. The Windhoek population was predominantly urban while in the Eastern Cape, Amatole Municipality which is largely rural is ranked among the poorest municipalities in South Africa [33]. In a study conducted by de Steenwinkel et al., (2008) in Mozambique, the reported prevalence of GBS was 1.8% in one of the poorest communities in Maputo which is much lower than the prevalence from this current study. The low prevalence of GBS was speculated to be linked to cultural norms and personal hygienic habits including male circumcision [34].

The prevalence of GBS in both Namibia and South Africa as shown in this study were not very different from those reported for other African countries such as Tanzania (23%), Uganda (28.8%) and Kenya (20.2%) [12, 29, 31]. The differences in the prevalence rates could be attributed to difference in study designs, prevalence at different gestational ages and different colonization rates in the different geographical regions. The prevalence rates in the different African regions do not show alignment to a particular region as the rates are varied.

Prevalence rates around the world vary between 10 and 40% [6] as the prevalence rates of GBS in Europe among pregnant women vary between 6.5 and 36% with more than a third of the studies reporting a prevalence of 20% or greater. Eastern Europe had a prevalence of 19.7–29.3%, Western Europe 11–21%, Scandinavia 24.3–36% and Southern Europe 6.5–32% [35] and they are similar to those recorded in African studies. The prevalence rates in both Namibia and South Africa are not very different from those in other parts of the world despite the differences in geographical location.

As shown in Tables 3 and 4, 27 (37.5%) study participants from Namibian had vaginal colonization and 13 (18.1%) had rectal colonization while 32 (44.4%) were simultaneously colonized vaginally and rectally. In the South African study subjects, 5 (13.5%) were vaginally colonized while 30 (81.1%) were dually colonized with GBS. In both populations, most of the women were dually colonized. In a similar study in Tanzania, colonization rates were 12.3% in the vagina and 5% in the rectum [12]. GBS colonises both the vagina and the rectum with colonization of either site resulting in vertical transmission to the baby during labour and or delivery while dual colonization of both the rectum and vagina increases the risk of vertical transmission to the new-born so the Centre for Disease Control (CDC) recommends rectovaginal sampling for detection of GBS.

GBS capsular types vary in different geographical locations. Capsular types Ia, II, III and V are the most prevalent around the world. Capsular type II was most prevalent in this study in both Namibia (59.4%) and South Africa (52.2%) as shown in Tables 5 and 6. Capsular types II, III and V constituted 94.1% of the Namibian isolates and 86.5% of the South African isolates in this study. These capsular types have been reported to be commonly associated with adverse pregnancy outcomes as well as neonatal morbidity and mortality [22] and they are predominantly the capsular types which have been isolated in other Southern Africa countries such as Zimbabwe and South Africa. However, a report from a study in South Africa in 2015 found a high prevalence of capsular types III (29.7%), Ia (25.8%) and V (10.9%) [30]. In a similar study in South Africa, Cools et al., (2016), reported a high prevalence of the same capsular types Ia (36.8%), V (26.3%) and III (14.0%).) while in Zimbabwe, Mavenyengwa et al., (2010) reported similar findings with the following prevalences: Ia (15.7%), Ib (11.6%), II (8.3%), III (38.8%) and V (24.0%) [18, 31]. Similar studies in Europe have equally reported capsular types II, III and Ia to be the most prevalent [35].

Capsular typing of GBS is important in determining the pathogenicity of the isolates and for epidemiological purposes. The capsular type distribution depends on factors such as geographical region, ethnicity or other characteristics of the population studied. Studies have shown variations in capsular type distribution of *S. agalactiae* when isolated from pregnant and non-pregnant women [36].

Even though the results of this study are similar to those of other African studies and are comparable to European countries, most of the women were dually colonised predominantly by capsular types commonly associated with adverse maternal outcomes and this poses a risk to both the mother and baby. However, the research was not able to follow up the outcomes of both the pregnant mothers and their babies.

## Conclusion

In this study, the prevalence of GBS in Namibia and South Africa was not different from other African countries and countries around the world. However, most of the pregnant women were both vaginally and rectally colonised with GBS which increases the risk of vertical transmission to the babies during delivery. The GBS capsular types predominant in pregnant women were the same capsular types prevalent in other African studies and commonly associated with adverse pregnancy outcomes.

## Abbreviations

AEMREG: Applied and Environmental Microbiology Research Group
CDC: Centre for Disease Control and Prevention
GBS: Group B Streptococcus
GMRDC: Govan Mbeki Research and Development Centre
IAP: Intrapartum Antibiotic Prophylaxis
LVS: Low Vaginal Swab
NUST: Namibia University of Science and Technology
RS: Rectal Swab
UFH: University of Fort Hare
USA: United States of America
